# Supplementary material for: Gunn-Hilsum Effect in Mechanically Strained Silicon Nanowires: Tunable Negative Differential Resistance
Source: Sci Rep. 2018 Apr 19;8:6273. doi: 10.1038/s41598-018-24387-y (PMC5908846; doi:10.1038/s41598-018-24387-y)
Supplement: Supplementary file 1 — Supplementary Information [file 41598_2018_24387_MOESM1_ESM.pdf]

Supplementary to Gunn-Hilsum Effect in Mechanically Strained Silicon Nanowires: Tunable Negative Differential Resistance

Daryoush Shiri[1], Amit Verma[2], Reza Nekovei[2], Andreas Isacsson[1], C. R. Selvakumar[3], and M. P. Anantram[4]

[1]Department of Physics, Chalmers University of Technology, SE-412 96 Göteborg, Sweden

[2]Department of Electrical Engineering and Computer Science, Texas A&M University-Kingsville, Kingsville, Texas 78363, USA

[3]Department of Electrical and Computer Engineering, University of Waterloo, Waterloo, Ontario N2L 3G1, Canada

[4]Department of Electrical Engineering, University of Washington, Seattle, Washington 98195-2500, USA

Calculation of first order electron-phonon scattering rate

In order to use the ensemble Monte Carlo (EMC) simulation, calculation of electron-phonon scattering rates is necessary. For each initial state starting from a given wave vector within the BZ, all possible final states in all possible sub bands with corresponding scattering rates for both phonon types (LA/LO) should be utilized. Both inter- and intra-sub band electron-phonon scattering events are calculated and tabulated to be used as input files for the EMC code.

a. Electron-LA phonon scattering

Figure A1 shows how an electron at the bottom of the indirect conduction band can scatter into many available secondary states within E_{Debye} window. If the rate of each scattering event is called $W(k_z, k_z', \tilde{\boldsymbol{q}})$, then the total scattering rate of the electron at k_z is found by summation over all available secondary states (k_z') and phonon wave vectors ($\tilde{\boldsymbol{q}}$) i.e.:

$$W_{k_z} = \sum_{k_z', \tilde{\boldsymbol{q}}} W(k_z, k_z', \tilde{\boldsymbol{q}}) = \sum_{k_z'} \sum_{\tilde{\boldsymbol{q}}} W(k_z, k_z', \tilde{\boldsymbol{q}}) = \sum_{k_z'} W(k_z, k_z') \quad \text{(A1)}$$

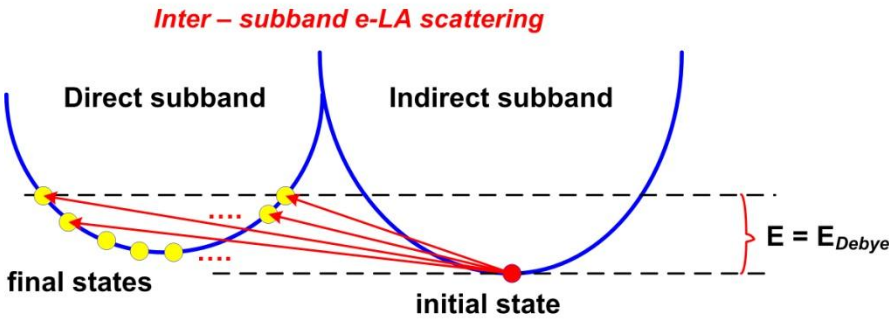

Figure A1. Inter-sub band electron-LA phonon scattering events starting from k_z.

Total momentum relaxation rate is found by the same equation in which individual rates, W, are weighted by a factor of (1 - k_z/k'_z). Derivations of momentum relaxation rates have been explained in detail in references [1,2]. Here we explain how the total scattering rate (W_{kz}) as well as each individual scattering rate [$W(k_z, k'_z,)$] are calculated for EMC simulation. Using Fermi's golden rule, the rate of a single scattering event can be written as follows where both momentum and energy are conserved and ψ corresponds to the mixed (electron and phonon) states.

$$W(k_z, k'_z, \tilde{\boldsymbol{q}}) = \frac{2\pi}{\hbar}\left|\langle\psi_{k_z}|H_{eP}|\psi_{k'_z}\rangle\right|^2 \delta(E(k'_z) - E(k_z) \pm \hbar\omega(\tilde{\boldsymbol{q}})).\, \delta_{q_z,\ k'_z - k_z} \qquad \text{(A2)}$$

The electron-phonon interaction Hamiltonian for phonons of LA type is given as:

$$H_{eP} = D\sum_{\bar{\boldsymbol{q}}} i|\bar{q}|\sqrt{\frac{\hbar}{2\rho V\omega(\bar{\boldsymbol{q}})}}\left(a_q e^{i\boldsymbol{q}.\boldsymbol{r}} + a_q^{\dagger} e^{-i\boldsymbol{q}.\boldsymbol{r}}\right) \qquad \text{(A3)}$$

where a_q and $a_q^{\dagger}$ are annihilation and creation operators. Since the z component of the phonon wave vector (q_z) is determined by conservation of momentum i.e. $q_z = k'_z - k_z$, the summation over phonon wave vectors spans all transversal components of phonon wave vectors. It is converted to integration according to:

$$\sum_{k'_z} \ldots \rightarrow \frac{L_z}{2\pi}\int \ldots dk_z = \frac{L_z}{2\pi}\int \ldots dq_z \;\text{ and }\; \sum_{\tilde{\boldsymbol{q}}} \ldots = \sum_{q_x, q_y} \ldots = \frac{A}{4\pi^2}\iint \ldots dq_x d\,q_y \qquad \text{(A4)}$$

where A denotes area of the nanowire. The area element for integration in equation A4 can be written in terms of radial (transversal) components of phonon momentum by converting the Cartesian coordinate to the polar one i.e. $dq_x dq_y = q_t dq_t d\varphi$. The angle, ϕ, changes from 0 to 2π. Using the same procedure as discussed in [2] the electron-LA phonon interaction Hamiltonian matrix element is reduced to equation A5 in which U stands for Bloch part of the electronic states.

$$\left|\langle U_{k_z}|H_{eP}|U_{k'_z}\rangle\right|^2 = \frac{D^2\hbar}{2\rho V}\frac{|\tilde{\boldsymbol{q}}|^2}{\omega_q}|S(|\tilde{\boldsymbol{q}}|)|^2 B_{\pm}(|\tilde{\boldsymbol{q}}|) \qquad \text{(A5)}$$

$B_{\pm}(|\tilde{\boldsymbol{q}}|)$ is the Bose-Einstein factor of phonons and it is $1/(e^{\frac{\hbar\omega_q}{K_B T}} - 1)$ for absorption and $1 + \frac{1}{(e^{\frac{\hbar\omega_q}{K_B T}} - 1)}$ for emission of a phonon. With transversal (radial) and longitudinal components of phonon wave vectors (q_t and q_z), the absolute value of phonon wave vector can be written as $|\tilde{\boldsymbol{q}}| =$

$\sqrt{q_t^2+q_z^2}$. The structure factor $S(|\tilde{\boldsymbol{q}}|)$ is defined as follows where *m* and *m′* are index of atoms in one unit cell. k_1 and k_2 denote the electron wave vector (momentum) of two different states and $\boldsymbol{r}_m$ is the coordinate of *m*'th atom.

$$S(|\tilde{\boldsymbol{q}}|) = \sum_m |C_{1m}(k_1)|^2 |C_{2m}(k_2)|^2$$
$$+ \sum_{m,m',m\neq m'} C_{1m}(k_1)\, C_{2m}^*(k_2) C_{2m'}^*(k_2) C_{1m'}(k_1) e^{-i\tilde{\boldsymbol{q}}\cdot(\boldsymbol{r}_{m'}-\boldsymbol{r}_m)} \quad \text{(A6)}$$

It is assumed that there is no overlap between the atomic orbitals of two neighboring unit cells i.e. the orbitals which belong to the same atom can have nonzero overlapping (interaction). The coefficients, C_{1m}, are the elements of the nanowire Eigen state ($N_{orbit} \times 1$ vector) at k_1 and as explained before they contain 10 numbers corresponding to orbitals of Si atom with index (*m*). Inserting equation A6 into equation A5 and using the results for equation A1 yields:

$$W_{k_z} = \frac{V}{(2\pi)^3} \cdot \frac{2\pi}{\hbar} \iiint |\tilde{\boldsymbol{q}}|^2 \frac{D^2\,\hbar}{2\rho V \omega(\tilde{\boldsymbol{q}})} B_\pm(|\tilde{\boldsymbol{q}}|). |S(|\tilde{\boldsymbol{q}}|)|^2$$
$$\times\, \delta(E(k_z') - E(k_z) \pm \hbar\omega(\tilde{\boldsymbol{q}})). \delta_{k_z,k_z'\pm q_z}. q_t dq_t d\varphi dq_z \quad \text{(A7)}$$

Using the linear dispersion of LA phonon we can write:

$$W_{k_z} = \frac{D^2}{8\pi^2\rho} \iiint \frac{q_t^2+q_z^2}{v_s\sqrt{q_t^2+q_z^2}} B_\pm(|\tilde{\boldsymbol{q}}|). |S(|\tilde{\boldsymbol{q}}|)|^2 . \delta(E(k_z') - E(k_z) \pm \hbar\omega(\tilde{\boldsymbol{q}})). \delta_{k_z,k_z'\pm q_z}. q_t dq_t d\varphi dq_z \quad \text{(A8)}$$

Further simplification of equation A8 results in:

$$W_{k_z} = \frac{D^2}{8\pi^2\rho v_s} \iiint q_t \sqrt{q_t^2+q_z^2} B_\pm(|\tilde{\boldsymbol{q}}|). |S(q_t,q_z,\varphi)|^2$$
$$\times\, \delta(E(k_z') - E(k_z) \pm \hbar\omega(\tilde{\boldsymbol{q}})). \delta_{k_z,k_z'\pm q_z}. dq_t d\varphi dq_z \quad \text{(A9)}$$

Since $|S(q_t,q_z,\varphi)|^2$ is the only term which depends on φ, therefore the integration over φ can be replaced by $\Phi(q_t,q_z)$ which is $\Phi(q_t,q_z) = \int_0^{2\pi} |S(q_t,q_z,\varphi)|^2 d\varphi$.

$$W_{k_z} = \frac{D^2}{8\pi^2\rho v_s} \iint q_t \sqrt{q_t^2+q_z^2} B_\pm(|\tilde{\boldsymbol{q}}|). \Phi(q_t,q_z) \times \delta(E(k_z') - E(k_z) \pm \hbar\omega(\tilde{\boldsymbol{q}})). \delta_{k_z,k_z'\pm q_z}. dq_t dq_z$$

(A10)

The precise calculation of scattering rate mandates large number of points in [0,2π] interval (N_{kz}>1000). Now the Dirac delta function can be manipulated as:

$$\delta\big(E(k_z') - E(k_z) \pm \hbar\omega(\widetilde{\boldsymbol{q}})\big) = \delta(E(k_z') - E(k_z) \pm \hbar v_s|\widetilde{\boldsymbol{q}}|)$$
$$= \delta\left(\hbar v_s(\frac{E(k_z')-E(k_z)}{\hbar v_s} \pm |\widetilde{\boldsymbol{q}}|)\right) = \frac{1}{\hbar v_s}\delta\left(\frac{E(k_z')-E(k_z)}{\hbar v_s} \pm |\widetilde{\boldsymbol{q}}|\right) \quad \text{(A11)}$$

where we have used δ(a*x*)=δ(*x*)/a. Replacing $|\widetilde{\boldsymbol{q}}|$ with $\sqrt{q_t^2 + q_z^2}$ and using Krönecker's delta which imposes $q_z = k_z' - k_z$ i.e.,

$$E(k_z') - E(k_z) = E(k_z \pm q_z) - E(k_z) = \Delta E_{kk'} \quad \text{(A12)}$$

we get,

$$W_{k_z} = \frac{D^2}{8\pi^2\rho v_s}\frac{1}{\hbar v_s}\iint q_t\sqrt{q_t^2 + q_z^2}.B_\pm(|\widetilde{\boldsymbol{q}}|).\Phi(q_t, q_z)\,.\,\delta\left(\frac{\Delta E_{uv}}{\hbar v_s} \pm \sqrt{q_t^2 + q_z^2}\right)dq_t dq_z \quad \text{(A13)}$$

The integration over q_t can be simplified more using $\delta(f(x)) = \frac{1}{|f'(x_0)|}\delta(x_0)$, where x_o is the single root of the function *f(x)* found by *f(x)=0*.

$$|\widetilde{\boldsymbol{q}}| = \sqrt{q_t^2 + q_z^2} = \pm\frac{\Delta E_{kk'}}{\hbar v_s} \rightarrow q_t = +\sqrt{(\frac{\Delta E_{kk'}}{\hbar v_s})^2 - q_z^2} \qquad \text{(Since } q_t > 0) \qquad \text{(A14)}$$

Equation A13 can be simplified further:

$$W_{k_z} = \frac{D^2}{8\pi^2\rho\hbar v_s^2}\iint q_t|\widetilde{\boldsymbol{q}}|B_\pm(|\widetilde{\boldsymbol{q}}|).\Phi(q_t, q_z)\,.\,\delta\left(\frac{\Delta E_{kk'}}{\hbar v_s} \pm |\widetilde{\boldsymbol{q}}|\right)dq_t dq_z \qquad \text{(A15)}$$

Recalling that $B_\pm(|\widetilde{\boldsymbol{q}}|)$ is only a function of $|\widetilde{\boldsymbol{q}}|$. Using equation A14 a relation between dq_t and $d|\widetilde{\boldsymbol{q}}|$ can be found as follows:

$$|\widetilde{\boldsymbol{q}}| = \sqrt{q_t^2 + q_z^2} \;\rightarrow d|\widetilde{\boldsymbol{q}}| = 2q_t.\frac{dq_t}{2\sqrt{q_t^2+q_z^2}} = \frac{q_t dq_t}{|\widetilde{\boldsymbol{q}}|} \rightarrow q_t dq_t = |\widetilde{\boldsymbol{q}}|d|\widetilde{\boldsymbol{q}}| \quad \text{(A16)}$$

Replacing $q_t dq_t$ according to equation A17 and using the sifting property of Dirac's delta function, equation A15 can be reduced to:

$$W_{k_z} = \frac{D^2}{8\pi^2\rho\hbar v_s^2}\int\left(\pm\frac{\Delta E_{kk'}}{\hbar v_s}\right)^2\left(B_\pm\left(\left|\pm\frac{\Delta E_{kk'}}{\hbar v_s}\right|\right)\right)\Phi\left(q_t = \sqrt{(\frac{\Delta E_{kk'}}{\hbar v_s})^2 - q_z^2}, q_z\right)dq_z =$$

$$\frac{D^2}{8\pi^2\rho\hbar^3 v_s^4}\int \Delta E_{kk'}{}^2\left(B_\pm\left(\left|\pm\frac{\Delta E_{kk'}}{\hbar v_s}\right|\right)\right)\Phi\left(q_t = \sqrt{(\frac{\Delta E_{kk'}}{\hbar v_s})^2 - q_z^2}, q_z\right)dq_z \quad \text{(A17)}$$

If we write the integration as a discrete summation over grid points along the 1D BZ, then rewriting equation A17 reveals how it is possible to single out individual rate ($W(k_z, k_z')$) between a pair of given states. Recalling that $\Delta q_z = \Delta k_z'$ we can write:

$$W_{k_z} = \sum_{k_z'} W(k_z, k_z') = \sum_{k_z'} \frac{D^2}{8\pi^2\rho\hbar^3 v_s^4}\Delta E_{kk'}{}^2 B_\pm\left(\left|\pm\frac{\Delta E_{kk'}}{\hbar v_s}\right|\right)\Phi\left(q_t = \sqrt{(\frac{\Delta E_{kk'}}{\hbar v_s})^2 - q_z^2}, q_z\right)\Delta k_z' \quad \text{(A18)}$$

b. Electron-LO phonon scattering

Similar to equation A1, the total electron-LO phonon scattering rate can be written as:

$$W_{k_z} = \sum_{k_z', \tilde{\boldsymbol{q}}} W(k_z, k_z', \tilde{\boldsymbol{q}}) \quad \text{(A19)}$$

The only difference is that individual scattering rate between k_z (at indirect conduction sub-band) and k_z' (at direct conduction sub-band), includes LO phonon with wave vector $\tilde{\boldsymbol{q}}$ and it is given by Fermi's golden rule similar to equation A2:

$$W(k_z, k_z', \tilde{q}) = \frac{2\pi}{\hbar}\left|\langle U_{k_z}|H_{op}|U_{k_z'}\rangle\right|^2 \delta(E(k_z') - E(k_z) \pm_a^e \hbar\omega_0) \quad \text{(A20)}$$

The electron-LO optical phonon interaction Hamiltonian matrix element is given as:

$$\left|\langle i|H_{op}|f\rangle\right|^2 = \frac{|D_{op}|^2\hbar}{2\rho V\omega_0}|S(|\tilde{\boldsymbol{q}}|)|^2 B_\pm(N(\hbar\omega_0) + \frac{1}{2} \pm_a^e \frac{1}{2})\ \delta_{k_z', k_z \pm q_z} \quad \text{(A21)}$$

Similar to the case of LA phonons, $S(|\tilde{\boldsymbol{q}}|)$, $B_\pm$ and ω_0 are structure factor, Bose-Einstein factor and frequency of dispersion-less LO phonon, respectively. Following the same procedure given for acoustic phonons, the summations in equation A19 can be simplified as follows:

$$W_{k_z} = \frac{|D_{op}|^2}{8\pi^2\rho\omega_0}\int_{q_z}\left(\int_0^{q_c} q_t\left[\int_0^{2\pi}|S(q_z, q_t, \phi)|^2 d\phi\right]dq_t\right).B_\pm\left(N(\hbar\omega_0) + \frac{1}{2} \pm_a^e \frac{1}{2}\right).\delta(F(k_z'))dq_z\ (or\ dk_z')$$

(A22)

The maximum allowable value of phonon transversal component within the BZ of bulk silicon which is q_c is equal to 1.9π/a [2]. For each value of q_z which is determined by the momentum difference of two consecutive states (i.e. $q_z = k'_z - k_z$), there are infinite allowed values for transversal component of **q** within [0, q_c] interval. F(k'_z) is the energy difference of initial and final states which is expressed as a function of k'_z i.e. $F(k'_z) = E(k'_z) - E(k_z) \pm_a^e \hbar\omega_0$. To further simplify the integration over q_z in equation A22 the following property of Dirac's delta function is used:

$$\delta\big(F(k'_z)\big) = \sum_{q_p} \frac{\delta(k'_z - q_p)}{\left|\frac{\partial F(k'_z)}{\partial q_z}\right|_{q_z=q_p}} = \sum_{q_p} \frac{\delta(k'_z - q_p)}{\left|\frac{\partial E(k'_z)}{\partial q_z}\right|_{q_z=q_p}} \quad \text{(A23)}$$

where $\frac{\partial F(k'_z)}{\partial k'_z} = \frac{\partial E(k'_z)}{\partial k'_z} = \frac{\partial E(k'_z)}{\partial q_z}$ and q_p are solutions of F(k'_z) =0 or in another word those final states (k'_z) which satisfy the energy conservation of $E\big(q_p\big) = E(k_z) \mp_a^e \hbar\omega_0$. Inserting right hand side of equation A23 into equation A22 yields:

$$W_{k_z} = \frac{|D_{op}|^2}{8\pi^2\rho\omega_0} \int_{q_z} \int_0^{q_c} \Phi(q_t, q_z) dq_t \, . B_\pm \left(N(\hbar\omega_0) + \frac{1}{2} \pm_a^e \frac{1}{2}\right) . \sum_{q_p} \frac{\delta(k'_z - q_p)}{\left|\frac{\partial E(k'_z)}{\partial q_z}\right|_{q_z=q_p}} . dq_z \quad \text{(A24)}$$

where $\Phi(q_t, q_z) = \int_0^{2\pi} |S(q_z, q_t, \phi)|^2 d\phi$. To single out the individual scattering rate i.e. $W(k_z, k'_z)$, the integration over q_z is written in its discrete form and noting that $\Delta q_z = \Delta k'_z$ we have:

$$W_{k_z} = \sum_{k'_z} W(k_z, k'_z) = \sum_{k'_z} \frac{|D_{op}|^2}{8\pi^2\rho\omega_0} \int_0^{q_c} \Phi(q_t, q_z) dq_t \, B_\pm \left(N(\hbar\omega_0) + \frac{1}{2} \pm_a^e \frac{1}{2}\right) . \sum_{q_p} \frac{\delta(k'_z - q_p)}{\left|\frac{\partial E(k'_z)}{\partial q_z}\right|_{q_z=q_p}} \Delta k'_z$$

(A25)

By interchanging summation over k'_z and q_p we have:

$$W_{k_z} = \sum_{k'_z} W(k_z, k'_z) = \sum_{k_p} \frac{|D_{op}|^2}{8\pi^2\rho\omega_0} \int_0^{q_c} \Phi(q_t, q_z) dq_t \, B_\pm \left(N(\hbar\omega_0) + \frac{1}{2} \pm_a^e \frac{1}{2}\right) \frac{1}{\left|\frac{\partial E(k'_z)}{\partial q_z}\right|_{k'_z=k_p}}$$

$$= \sum_{k_p} W(k_z, k_p) \quad \text{(A26)}$$

k_p are those values of secondary state wave vectors (k'_z) which satisfy the F(k'_z) =0. Hence corresponding to each k_z (initial state) there are a few secondary states (k_p) to which electron can scatter by absorbing or emitting a LO phonon. And the total rate is simply found by adding each individual term according to equation A26. Albeit the derivative of $E(k'_z)$ can be calculated either

numerically using finite difference scheme or using analytic derivation assuming effective-mass theory i.e. assuming that each sub band is of parabolic type with a defined effective mass. Both methods return the same values of scattering rates.

c. Post processing of scattering data for EMC simulation

The electron-LA phonon and LO phonon scattering rates (both inter- and intra-sub band) are implemented in MATLAB according to equation A18 and equation A26, respectively. Figure A2 shows the algorithm or pseudo code of saving individual electron-LA phonon scattering rates for initial states within the first sub band (S_1). The same method can be generalized to the cases where initial states are in second sub band (S_2), 3rd sub band (S_3), and 4th sub band (S_4).

```
FOR i=1:N_kz      % (N_kz : number of grid points along BZ)
   find available states within  E_i - E_debye ≤ E_i ≤ E_i + E_Debye
   2nd-k-indices(i,:)=k'_z indices
END
FOR i=1:N_kz
  read E_i,b1, ψ_i,b1, k_i,b1
  FOR j=1:length_of (2nd_k_indices(i,:))
        read k_z_indx=2nd-k-indices(i,j)
        IF k_z_indx(j) ϵ band1
             save ψ_j,b1 and E_j,b1 and k_j,b1
        ELSE
             save ψ_j,b2 and E_j,b2 and k_j,b2
        END
        Calculate W(k_i,b1,k_j,bx)  x ϵ {b1,b2}    EQ. A18
        IF E_i,b1>E_j,bx   (LA emission)
            Indiv_emit(i,j)= W
        ELSE   (LA absorption)
            Indiv_absorb(i,j)= W
       END
  END
END
Grouped_abs_emit ←CALL indiv-absorb-emit-sorter
Total_scatt_rate=sum_of_columns(Grouped_abs_emit)   (full EQ. A18)
Plot(k_zi, Total_scatt_rate)
```

Figure A2. Flowchart of saving individual rates for e-LA phonon scattering events.

The MATLAB code which implements this algorithm groups the individual emission/absorption rates and adds them together. Decision is made based on the number of k_j (secondary state indices) which

determines if the secondary state belongs to *band1* (intra-sub band) or *band2, 3 and 4* (inter sub band). The concept of the grouping of individual absorption and emission rates and sorting them according to inter- or intra-sub band scattering, were shown in Figure 2 of the manuscript. The algorithm for this decision making is shown in Figure A3. The same algorithm of Figure A3 is applicable to the case of electron-LO phonon scattering (using equation A26) but special care is required when secondary states are saved in the first loop of Figure A2.

```
FOR i=1:Nkz (each initial state in band1)
    AB=inidv_abs(i,:), EM=indiv_emit(i,:);
    2nd_k_index=2nd_k_indices(i,:);
END
FOR each pair of (AB,EM) vectors DO
Make AB and EM to be the same size
FOR m=1:size(2nd_k_index)
        IF 2nd_k_index ϵ band1
            Z(m)=1
         ELSE
            Z(m)=2
         END
END
ABS11=ABS12=0; EM11=EM12=0;   % initializing
FOR m=1:size(AB)
        IF Z(m)=1
            ABS11=ABS11+AB(m);
        ELSE
            ABS12=ABS12+AB(m);
        END
END
REPEAT the previous loop for EM
END
Grouped_abs_emit=[ ABS11; EM11; ABS12; EM12]
GO to line#1 and REPEAT the same for (each initial state in band2)
```

Figure A3. Algorithm of grouping and sorting individual LA phonon absorption and emission rates.

In contrast to LA phonons which have a continuum of energies from 0 to E_{Debye}, the LO phonons have all a constant energy of E_{LO}=63 meV. Thus if the criteria to choose the secondary state is set to be E_i - 63meV ≤ E_i ≤ E_i + 63meV, there may be cases with no phonon due to coarse grid of k_z axis. On the other hand by choosing these criteria of E_i - 63meV±Tol ≤ E_i ≤ E_i + 63meV±Tol, it is possible to find many secondary states close to each other within the tolerance window. Resolving this problem is done by selecting a unique index among many closely spaced indices. For example if the indices of

available secondary states which are sorted in a vector are something like K_{index} = [17 18 19 25 33 34 35 90 171 172 173 174 175 233], then the output must be K_{select} = [18 25 34 173 233].

References:

[1] M. Lundstrom, *Fundamentals of Carrier Transport*, Cambridge University Press, Cambridge, (2000).

[2] A. K. Buin, A. Verma, and M. P. Anantram, *Carrier-phonon interaction in small cross-sectional silicon nanowires*, J. Appl. Phys. 104, 053716, (2008).
